# Commentary: Weighty data: importance information influences estimated weight of digital information storage devices

**DOI:** 10.3389/fpsyg.2016.00709

**Published:** 2016-05-10

**Authors:** Anna M. Borghi

**Affiliations:** ^1^Department of Psychology, University of BolognaBologna, Italy; ^2^Institute of Cognitive Sciences and Technologies, CNRRome, Italy

**Keywords:** weight, embodied cognition, grounded cognition, modularity of perception, distributional theories

In three well-designed experiments, Schneider et al. (2014) demonstrate that the importance ascribed to the content influences weight perception of a USB or of a data-storage device. I will briefly discuss the theoretical implications of these results for the recent debate on “penetrability” of perception and then more generally for embodied and grounded views of cognition; finally I will argue that it is important to study weight.

*A. Penetrability of perception*. The weight perception of a device is influenced by the estimated importance and self-relevance of the data it contains: the effect found clearly contradicts the view, according to which perception is an independent module and cannot be “penetrated” by cognition (Vinson et al., forthcoming). However, the method used by the authors has a major limitation that might lead to possible critiques by scientists who favor a modular view of perception: they could namely argue that the interaction of relevance with weight codes occurs at a post-perceptual level.
  This limitation lies in the choice to use explicit estimates instead of a task involving a bodily action. The limitation is partially recognized and discussed by the authors, and can lead to further research. The authors decided to use explicit estimates of weight, claiming “We assume that they report their physical experience of weight.” This assumption is questionable in light of the fact that, in many domains, it has been shown that explicit estimates are not always coherent with implicit measures: for example, people overestimate their ability to reach for objects (Fischer, 2005). Given that weight is an embodied property, I think that in their future research the authors could use more implicit tasks, and possibly tasks involving the body. Demonstrating that cognition influences not only explicit estimates, but implicit motor behaviors, for example the way to grasp an object, would be stronger and could fortify the theoretical implications of the results.
  Some years ago we demonstrated using an “implicit” motor task that a high level cognitive process, language, influences weight perception (Scorolli et al., 2009). Participants listened to sentences referring to light vs. heavy objects (e.g., “pillow” vs. “chest”), then they were required to lift one of two visually identical boxes, a heavy and a light one. The kinematics of the lift delay, i.e., of the time immediately following the object grasping, was different: when the weight implied by the sentence and the weight of the box corresponded (e.g., heavy–heavy), participants' time delay was larger. This suggests that the simulation of the weight formed during language comprehension occupied resources and, interfering with the concurrent motor task, determined a processing delay.
  *B. Embodied and grounded views*. Two aspects make this study an important one for embodied and grounded views of cognition, i.e., for the views according to which cognition is shaped by our body and by situated experiences (e.g., Barsalou, 2008).
  The first is that it demonstrates that even abstract concepts are grounded in sensorimotor system. As argued by the authors, bits or bytes are unphysical entity, but people easily provide estimates of their weight, and these estimates vary across contexts (see Borghi and Binkofski, 2014, for an overview of recent embodied theories on abstract concepts).
  The second is that the results hold both for information that can be considered as objectively important and for information that is important for oneself.
  Even though I really like this work, I think that, if seen from an embodied and grounded perspective, the current study has at least one limitation. As they currently are, the results of the present paper favor a grounded approach to cognition but are compatible also with a distributional view of meaning, according to which the effect could be due to an associative relationship between importance and weight. While it is possible that both embodied processes and linguistic associations do play a role in accounting for the effect, it would be important to rule out that the hypothesis that the results are due *only* to linguistic associations. The conceptual metaphor is likely grounded in sensorimotor processes, in some motor-based association between weight and importance. However, it remains an open issue whether the effect is due to the long-term influence of cultural metaphors associating weight and importance, or to the online building of a simulation. More crucially, whether the effect found is entirely based on sensorimotor information, whether it emerges from linguistic associations or whether both embodied and linguistic experience play a role is obviously an empirical matter, and it should be tested.
  *C. The role of weight*. A final reason why I like this study is that it focuses on weight. Weight is a peculiar property: differently from visually detectable properties such as shape and size, it cannot be fully determined with the eyes. When we watch someone lifting a weight, we can infer how heavy it is. However, when we simply see an object we can form expectations but we really learn its weight only interacting with the object. To some extent, weight is really an “embodied” property (Morlino et al., 2015), and the very fact that cognition influences such an “embodied” properties is remarkable.

I would like to conclude arguing that to study weight is important, also due to the fact that so far weight has not yet received much attention when compared to properties such as shape or color. Lightness and heaviness were appreciated by writers: Milan Kundera wrote about the unbearable lightness of being; Italo Calvino upheld the values of lightness, clarifying that his working method involved the subtraction of weight. Lightness and heaviness are important properties for psychologists and cognitive (neuro)scientists. Studying what it means to perceive objects and entities as light and heavy, both in literal and in metaphorical terms, is really worth of further investigation for people who are interested both in how cognition is shaped by the body, and in how our perception and interaction with the world is influenced by cognition.

## Author contributions

The author confirms being the sole contributor of this work and approved it for publication.

### Conflict of interest statement

The author declares that the research was conducted in the absence of any commercial or financial relationships that could be construed as a potential conflict of interest.

## References

[B1] Barsalou L. W. (2008). Grounded cognition. Annu. Rev. Psychol. 59, 617–645. 10.1146/annurev.psych.59.103006.093639 17705682

[B2] Borghi A. M. Binkofski F. (2014). Words as Social Tools. An Embodied View on Abstract Concepts. New York, NY: Springer.

[B3] Fischer M. H. (2005). Perceived reachability: the roles of handedness and hemifield. Exp. Brain Res. 160, 283–289. 10.1007/s00221-004-2007-x 15351926

[B4] Morlino G. Gianelli C. Borghi A. M. Nolfi S. (2015). Learning to manipulate and categorize in human and artificial agents. Cogn. Sci. 39, 39–64. 10.1111/cogs.12130 25041751

[B5] Schneider I. K. Parzuchowski M. Wojciszke B. Schwarz N. Koole S. L. (2014). Weighty data: importance information influences estimated weight of digital information storage devices. Front. Psychol. 5:1536. 10.3389/fpsyg.2014.01536 25620942 PMC4287016

[B6] Scorolli C. Borghi A. M. Glenberg A. M. (2009). Language-induced motor activity in bimanual object lifting. Exp. Brain Res. 193, 43–53. 10.1007/s00221-008-1593-4 18925389

[B7] Vinson D. W. Abney D. H. Amso D. Chemero A. Cutting J. E. Dale R. (forthcoming). Perception, as you make it. Behav. Brain Sci. 10.1017/S0140525X15002678 28355860

